# Circulating tumor cells: Blood-based detection, molecular biology, and clinical applications

**DOI:** 10.1016/j.ccell.2025.07.008

**Published:** 2025-07-31

**Authors:** Charles S. Dai, Avanish Mishra, Jon Edd, Mehmet Toner, Shyamala Maheswaran, Daniel A. Haber

**Affiliations:** 1 Krantz Family Center for Cancer Research, Mass General Brigham Cancer Institute and Harvard Medical School, Boston, MA 02114, USA; 2 Center for Engineering in Medicine and Surgery, Massachusetts General Hospital and Harvard Medical School, Boston, MA 02114, USA; 3 Shriners Children’s Hospital, Boston, MA 02114, USA; 4 Howard Hughes Medical Institute, Chevy Chase, MD 20815, USA

## Abstract

Circulating tumor cells (CTCs) are cancer cells, shed from primary tumors or metastases into the bloodstream. The first non-invasive “liquid biopsy” for cancer monitoring, CTCs have been largely surpassed by circulating tumor DNA (ctDNA) for clinical applications, given the ease of DNA sequencing without specialized cell isolation methods. However, emerging rare cell capture technologies that can process larger blood volumes and enable advanced single-cell analyses may enhance the range and potential of CTC-based biomarkers. CTCs are increasingly valuable for assessing tumor heterogeneity, guiding protein biomarker-driven cancer immune therapies, and assessing heterogeneous drug resistance, as well as for detecting minimal disease. CTCs, thus, remain central to understanding cancer dissemination and are poised to offer complementary diagnostic roles in the application of minimally invasive liquid biopsies for cancer. Here, we review recent advances in the study of these rare circulating cancer cells and discuss current limitations and future directions.

## INTRODUCTION

Genetically targeted therapies and immunotherapies have transformed the treatment of many cancers, but acquired drug resistance and lethal metastatic disease remain a major challenge. Hematogenous dissemination of metastases is mediated by CTCs that travel from the primary tumor to multiple distant organs, where they can establish new cancerous lesions and then spread to additional organ sites.^1-3^ In addition to their importance to understanding mechanisms of metastasis, CTCs raise interesting therapeutic and diagnostic questions: for instance, can understanding the molecular features of CTCs, including factors that enable their intravasation, their survival in the bloodstream, and their extravasation to generate distant lesions, inspire novel therapeutic strategies to suppress cancer metastasis? Can CTCs also be utilized to non-invasively interrogate molecular markers of progression during treatment, improve risk stratification prior to initiating therapy, or even possibly diagnose early cancerous lesions before the development of viable metastases? While we have learned much about the biological properties of CTCs, their clinical promise has been limited by technological challenges in isolating such extraordinarily rare cancer cells, which are surrounded by far more numerous normal blood components. Comparatively, simpler DNA sequencing-based strategies utilizing cell-free circulating tumor-derived DNA (ctDNA) have emerged as the more clinically useful “liquid biopsy”, enabling identification of cancer-associated mutations linked to drug resistance or early detection of cancer through DNA-based signatures.^4^

Innovation in both cancer diagnostics and therapeutics, however, are obligate companions: novel therapies often require relevant diagnostics for appropriate patient selection and monitoring, while novel diagnostics have limited utility unless they inform therapeutic choices. Accordingly, the advent of cancer immunotherapies and protein-targeted antibody-drug conjugate or T cell-based therapies have recently stressed the relevance of epitope assessment on the tumor cell surface to help guide treatment response. Such biomarkers are readily assessed via tumor cell-based analytics.^5-9^ Furthermore, therapeutic responses that induce transcriptional changes in cancer cell state or lineage, or those that induce heterogeneous subpopulations of refractory tumor cells, can be characterized via single cell-based biomarkers at a highly granular level.^10-15^ The ability to detail single-cell clonal heterogeneity raises the possibility of applying these findings toward predicting treatment resistance or guiding sequential therapy. Finally, the combination of DNA, RNA, and protein analytes derived from intact cells in the blood could enable a definitive diagnosis of cancer in patients without an invasive tissue biopsy, or may reveal functionally actionable subclonal mutations not evident from single lesion biopsy-based analysis.^16-18^ These current and emerging clinical scenarios have introduced new avenues for deploying ctDNA and CTC-based biomarkers as complementary liquid biopsy technologies. While ctDNA-based assays are readily available commercially and have played an increasingly important role in molecular monitoring of cancer, the technological challenges in rare cell enrichment have limited the clinical application of CTC-based analyses. Yet, recent methodological advances that permit interrogation of larger blood volumes, containing a higher number of CTCs and combined with sophisticated molecular analytics, may provide new opportunities to redefine CTCs in research and clinical practice. The enrichment of greater numbers of CTCs, using cell isolation platforms that are easier to deploy, will likely enable specific clinical applications addressing the biological features of heterogeneous disease, along with patient stratification for cancer immunotherapies.^14,15,19-23^ Similarly, future applications in early cancer detection and molecular characterization of minimal residual disease may complement current ctDNA-based analyses. This review builds on previous technological, molecular, and clinical reviews on CTCs,^4,24-35^ focusing on new directions likely to impact our understanding and treatment of cancer over the coming years.

## RARE CANCER CELLS IN THE BLOOD

In 1869, Thomas Ramsden Ashworth first described the observation that microscopic tumor cells were present in the blood of a man with metastatic cancer.^36^ Since then, CTCs have been detected across a breadth of malignancies of both epithelial and non-epithelial origin.^37-47^ It has been estimated that millions of CTCs are shed daily into the circulation per gram of tumor tissue (representing as much as ~0.1–1 billion cancer cells).^48-50^ However, CTCs still comprise only a minute fraction of total cells present in blood, with as few as one tumor cell per 10 billion blood cells.^21,51^ On average, current commercial technologies can only identify 1–10 CTCs in a standard 7.5 mL tube of blood from roughly 50–80% of patients with advanced metastatic cancer.^51^

CTCs have been classically defined as nucleated cells in a blood sample that stain negative for the ubiquitous leukocyte marker CD45, but positive for epithelial cytokeratins.^37,52,53^ While useful, this definition may also raise some diagnostic uncertainty. A significant number of cells in circulation stain for both CD45 and cytokeratin, a finding that has largely been attributed to spurious staining artifacts, including antibody adherence to the surface of some leukocytes.^51,54^ Recent evidence has emerged suggesting that some tumor cells might form true “hybrids” with leukocytes, potentially with altered functional properties,^55-59^ a provocative set of findings that warrant further molecular studies. On the other hand, a few cells in the blood may stain neither for CD45, nor for epithelial cytokeratins.^11,15,60-63^ These include CTCs from non-epithelial malignancies such as sarcomas and melanomas, or epithelial cancers that have undergone various degrees of epithelial-to-mesenchymal plasticity (EMP, EMT)—prompting the use of other lineage-related CTC markers.^11,44,45,60,64-67^ Indeed, in blood specimens from patients with advanced prostate cancer, small “double-negative” cells completely lacking in CD45 and cytokeratin staining were recently found to represent neuroendocrine lineage transformation, confirmed by the presence of neuronal markers and chromosomal aneuploidy (copy number variation; CNV).^15^ Thus, the presence of CNV, identified at the single-cell level, could ultimately provide a *bona fide* definition of CTCs, as cells bearing CNV can be readily distinguished from atypical non-hematopoietic cells without aneuploidy, and circumvent the risk of staining artifacts in CTC identification. Of note, some non-hematopoietic cells without CNV may represent benign epithelial cells in circulation; while relatively uncommon, such cells may be more prevalent in pre-cancerous or inflammatory conditions.^68,69^ Similarly, rare endothelial cells may also be present in the blood,^70^ but are not typically enriched using current CTC isolation platforms.^15^

**Figure 1.**
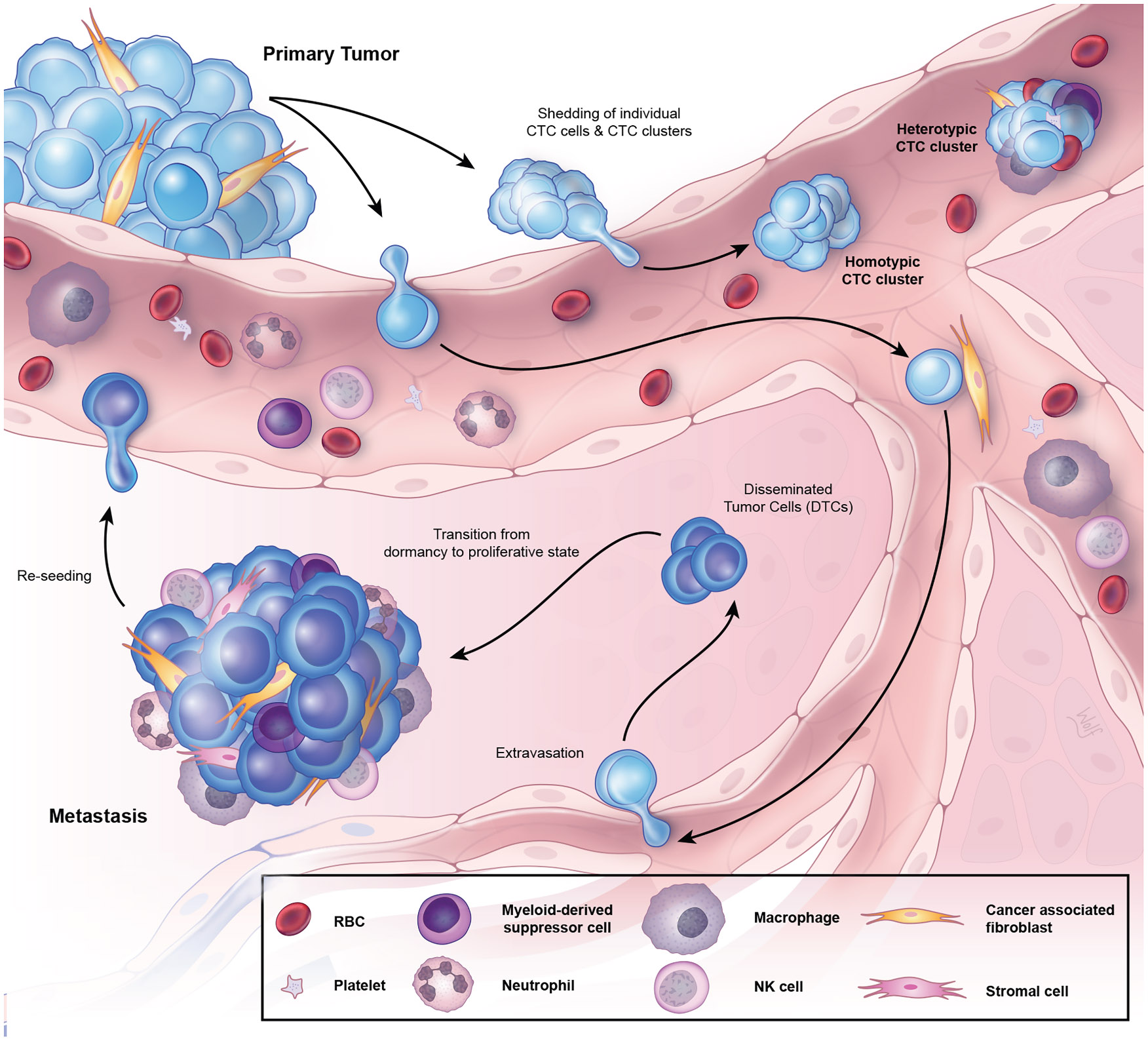
Circulating tumor cells in the metastatic cascade CTCs are shed from the primary tumor as either single cells or clusters, which can affiliate with hematopoietic cells in circulation. Upon extravasation into tissues, DTCs form the basis of early lesions with metastatic potential. DTCs often require additional stromal support to progress into clinically overt metastatic lesions, which can fuel re-seeding of CTCs in the circulation. Illustration by Nicole Wolf, MS, ©2025.

As noted in greater detail below, some CTCs travel in clusters, ranging from doublets to dozens of cancer cells tethered together.^71,72^ While most clusters are restricted to tumor cells, some may include tumor-derived fibroblasts, blood-derived leukocytes, or other cells^28,29,32,73,74^ (Figure 1). Very large clusters may comprise “microemboli” that are visible histologically in tissue sections as they occlude blood vessels.^75-78^ However, smaller clusters appear to travel unimpeded through capillary beds; within bioengineered models CTC clusters can align as a single row as they transverse narrow lumens, only to reassemble as a three-dimensional cluster upon exit.^79^ This phenomenon may facilitate their ability to effectively circulate in the blood and extravasate at distal sites. Microscopic imaging and scoring of CTCs stained with antibodies against relevant tumor-specific epitopes has been key to their definition but may also limit clinical application. Advanced fluorescence-based microscopy is not readily available within most clinical pathology labs, and even standard FDA-approved platforms, such as CellSearch, require extensive training of personnel to manually validate candidate CTCs identified through automated imaging.^80^ More sophisticated AI-based image analysis will undoubtedly enhance throughput, although high-quality imaging of cells stained for multiple epitopes and processed through complex enrichment procedures will continue to require advanced microscopic capabilities.^65,81-83^ Alternatively, RNA-based technologies to detect lineage markers in CTC-enriched products could help automate high-throughput molecular quantitation of CTC burden. For example, early studies in melanoma focused on tumor-specific transcripts using RT-PCR analysis applied to all mononuclear cells in blood,^84,85^ and more recent reports have demonstrated successful droplet-based digital PCR quantification (ddPCR) of highly restricted lineage-specific transcripts from breast, liver, melanoma and prostate CTC-enriched cell populations.^86-90^ Additional RNA-based studies of blood cell populations enriched for CTCs, without single-cell isolation, have allowed for quantitation of the androgen receptor (AR) splice variant AR-V7 in prostate CTCs, which predicts for resistance to AR pathway inhibitors.^88,91-93^ They have also enabled non-invasive pharmacokinetic assays to measure downregulation of transcripts associated with estrogen receptor (ER)-signaling in CTCs from patients with breast cancer treated with hormonal therapies.^87^ More recently, long-range nanopore sequencing has also been applied to characterize hypomethylated domains of DNA within CTCs, as a potential tool for the detection of localized prostate cancer.^94^ The development of progressively sophisticated imaging and molecular readouts, increasingly at the single-cell level, are now poised to generate unique information to inform cancer diagnostics. However, the limiting input of tumor material —namely, the very small number of individual CTCs present within a standard tube of blood— remains a critical bottleneck for robust clinical applications. Given this major challenge, interrogation of larger blood volumes using novel technologies is likely necessary to improve upon the utility of CTC-based diagnostics.^14,15,19,21-23,80^

## CTC ENRICHMENT TECHNOLOGIES

**Figure 2.**
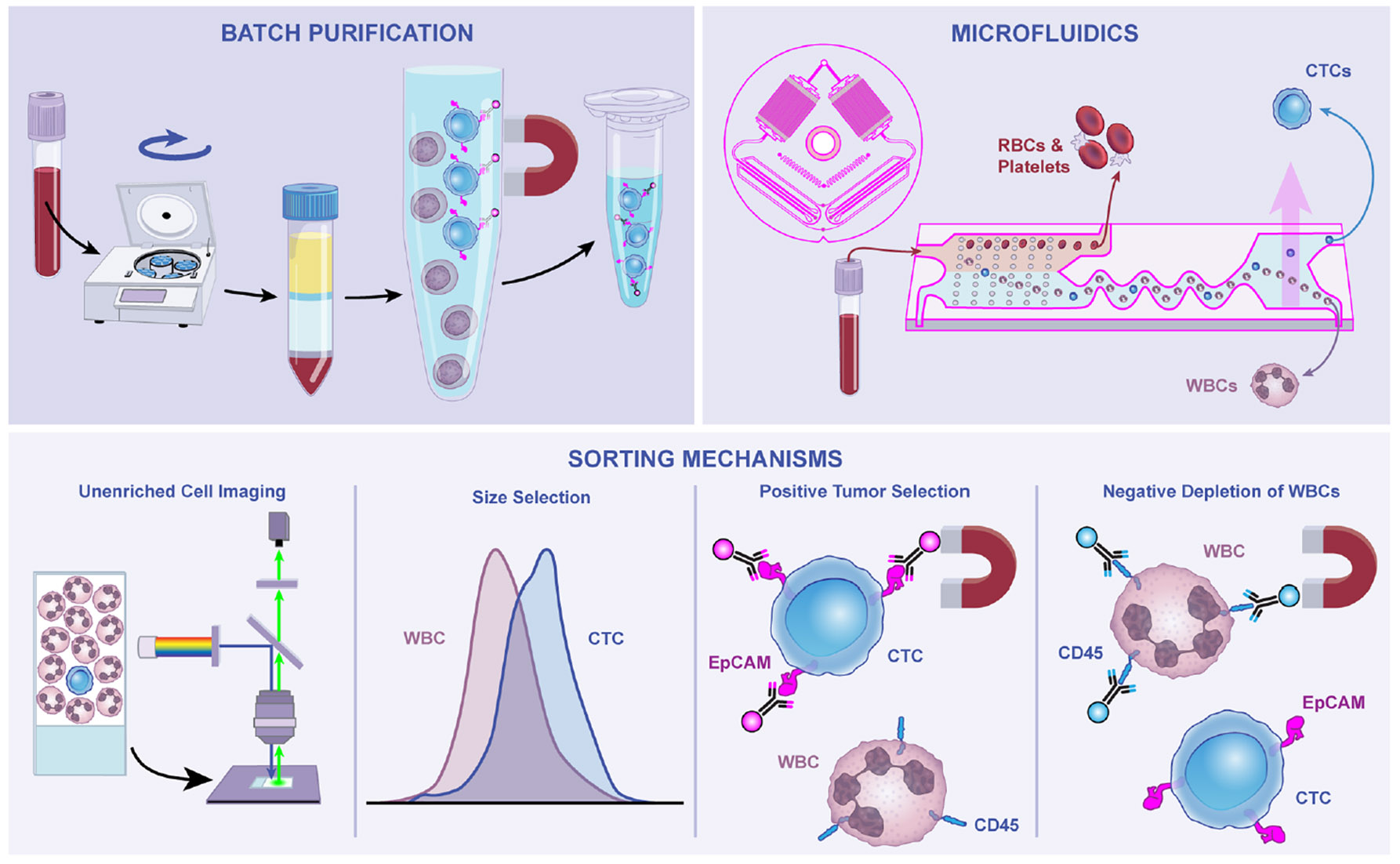
Technological advances to purify circulating tumor cells from blood Principles of CTC enrichment generally rely upon either batch purification or microfluidics to debulk hematopoietic cells and permit efficient capture of target CTCs. Additional modalities, including imaging-based technologies, size-based capture, and epitope selection of CTCs or WBCs, are often additionally required to achieve sufficient purity for downstream molecular applications. Illustration by Nicole Wolf, MS, ©2025.

Perhaps more than other traditional diagnostic tests for cancer, the isolation of CTCs depends upon complex technical processes and diverse methods that enable their distinction from surrounding blood cells (Figure 2). Central to this complexity is the extraordinary level of purification needed to isolate CTCs from phenotypically similar white blood cells (WBCs).^15^ The level of cell enrichment required is dependent on the sensitivity of downstream CTC analyses. While ddPCR-based quantitation of tissue-specific transcripts or immunostaining-based imaging is compatible with moderate levels of 10^3^- to 10^4^-fold enrichment,^86-89^ single-cell “-omics” analyses often require >10^8^-fold enrichment. As such, the ideal CTC enrichment strategy must be aligned with the intended analytical application. With this in mind, we note that two main classes of enrichment strategies exist: methods that achieve up to 10^4^-fold CTC enrichment and single-cell isolation approaches that additionally enhance cumulative purity of up to 10^8^-fold for more stringent downstream applications. In general, CTC isolation strategies exploit one or more distinct molecular, immunological, or biophysical properties that are thought to be unique to CTCs. We focus on some fundamental principles but also refer the reader to other technology-focused reviews.^31,95^

### Direct visualization of CTCs

Perhaps the least biased CTC scoring technology has been developed by Epic Sciences, involving initial lysis of red blood cells (RBCs), followed by plating of all remaining blood cells on multiple large slides that are then stained for the presence of epithelial markers.^96,97^ AI-based analytic software is then deployed to scan many millions of cells for those harboring features of CTCs. One advantage of this technology and similar platforms^98^ is that it circumvents the need for initial cell enrichment, but it also suffers from scalability and difficulty in deploying molecular tools beyond imaging to assess candidate CTCs surrounded by massive numbers of hematopoietic cells. Background staining presents a significant challenge in unpurified blood cell analyses, especially when CTCs are present at lower frequencies (<5 CTCs/10 mL blood); hence most platforms require batch or microfluidic enrichment of potential CTCs prior to imaging analysis.

### CTC capture based on size or physical properties

CTCs are generally believed to be larger than most hematopoietic cells, which can be exploited using microfiltration enrichment technologies^99^ and microfluidic size-based separation.^100,101^ The Parsortix PC1 system was recently FDA cleared for CTC capture in patients with metastatic breast cancer.^102,103^ In addition, density gradient centrifugation, inertial size separation, and other size-based separation technologies have been deployed to enrich for CTCs.^104^ Alternative approaches have focused on higher membrane capacitance and lower cytoplasmic conductance compared with leukocytes,^105^ as well as dielectric properties,^106,107^ deformability,^108-110^ or density.^111^ However, these techniques often rely on the larger size of CTCs as a principal property for separation.^31^ While these technologies avoid epitope-based bias, recent studies have revealed that CTCs overlap considerably in size with WBCs.^15,63^ Thus, size-based selection alone may lead to substantial CTC loss and result in poor enrichment rates in cancers where CTC size is not sufficiently distinct from that of WBCs. Alternative technologies take advantage of microfluidic size-based sorting approaches to selectively deplete much smaller blood components (RBCs and platelets), without relying on size differences to distinguish between WBCs and CTCs.^112-114^

### Positive immunoselection of CTCs

Immunoselection is one of the most widely used methods for enriching CTCs. This involves two common approaches: positive selection utilizing cell surface markers present on CTCs, or negative selection to remove contaminating WBCs by targeting common leukocyte proteins (Figure 2). For positive selection, the commonly expressed epithelial transmembrane glycoprotein EpCAM has been widely utilized.^115^ The CellSearch platform, which is FDA-cleared and standardized for enumerating CTCs for clinical prognostication in advanced cancers, relies upon magnetically conjugated antibodies against EpCAM to separate CTCs from surrounding blood cells using batch purification.^37^ More recently, this platform has been complemented by an array of compatible single-cell sorting technologies.^116-122^ However, the efficiency of CellSearch is currently limited by relatively high target cell losses from the washing and centrifugation steps via batch purification to achieve magnetic-based enrichment. Microfluidic approaches have been deployed to limit losses by combining magnetic sorting through continuous flow channels with capture using EpCAM antibody-coated structures.^39,65^ However, EpCAM-based capture has the disadvantage of relying on an epithelial marker not present in some tumors and which may be lost as cells undergo EMT.^11,123,124^ Alternative approaches for positive immunocapture have relied upon lineage-specific cell surface markers or common cancer epitopes, including folic acid receptor,^125,126^ PSMA,^66,127,128^ EGFR family proteins,^129^ S100,^130^ or other tumor/lineage-specific cell surface markers. Nevertheless, these strategies retain the inherent bias of pre-identifying a tumor marker of interest.

### Depletion of hematopoietic cells to achieve negative enrichment of CTCs

An alternative, less-biased enrichment strategy involves depleting well-characterized blood cell components, thereby enriching for untagged CTCs^63,112-114^ (Figure 2). These so-called “negative depletion” technologies employ antibodies targeting common epitopes on all WBCs, rather than variable markers on tumor cells, but they do often require multi-modal strategies^131^ or ultra-efficient microfluidics to effectively deplete hematopoietic cells.^15,21,112^ Among the most effective are microfluidic platforms that utilize geometric effects, wall lift forces, and microfluidic flow to deplete RBCs and platelets, while ordering nucleated cells (WBCs and CTCs) into a single flow path—from which strong magnetic forces can deplete magnetically tagged WBCs, leaving untagged, potentially viable CTCs in the collection channel.^95,113,114,132^ Such platforms can achieve 0.01–1% purity of CTCs,^15,21,112^ which can be further enriched using downstream positive selection strategies via selected cancer-specific markers. Microfluidic technologies are particularly gentle in sorting rare cells, facilitating subsequent culture of single CTCs or CTC clusters,^99,133,134^ as well as single-cell molecular characterizations.^15^

### Considerations for optimizing rare cell capture for single-cell analyses

**Figure 3.**
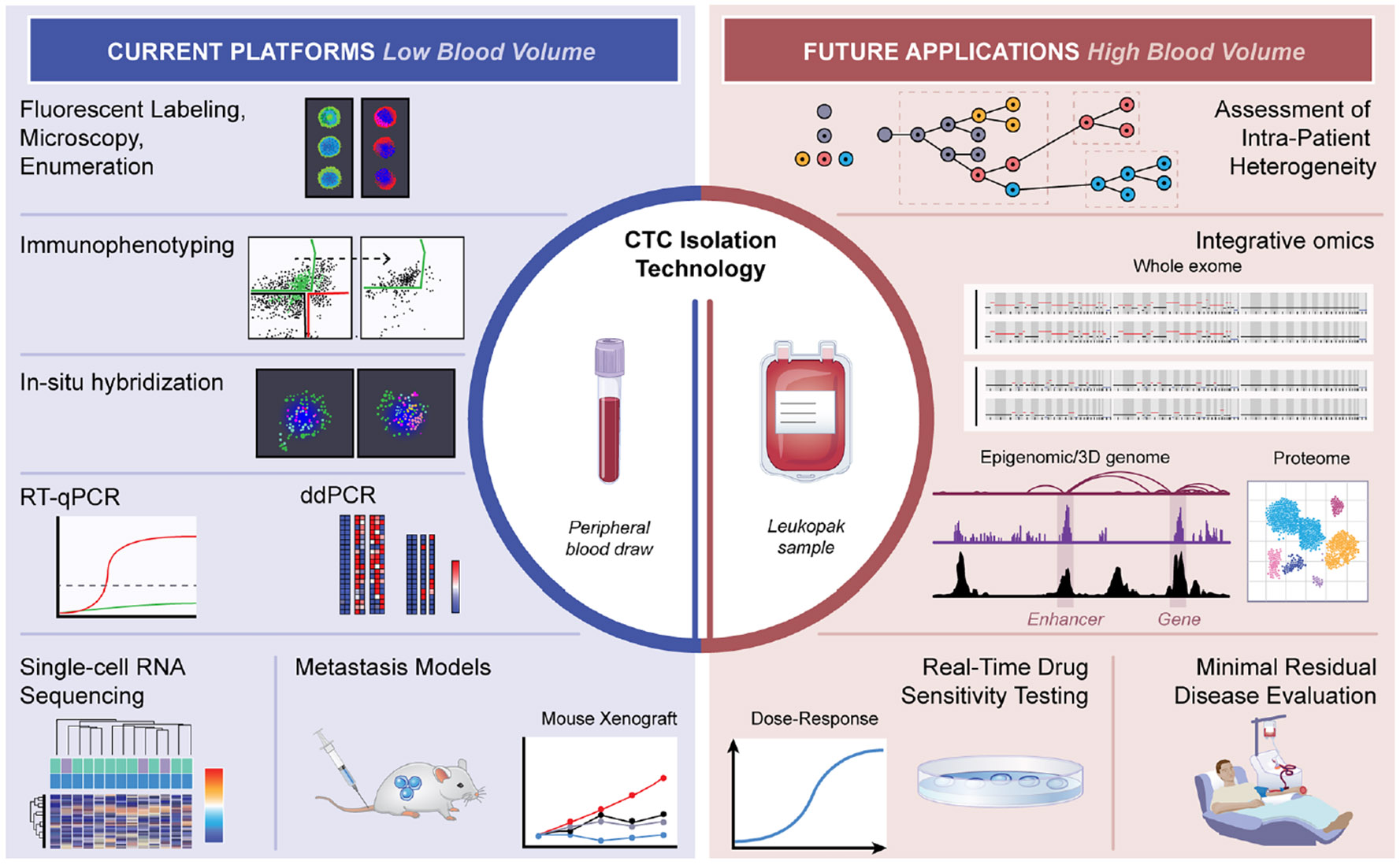
Low and high blood volume applications for circulating tumor cells While many molecular applications currently employ CTC enrichment technologies that are limited by rare CTCs in peripheral blood, advances in high volume blood interrogation have the potential to expand upon the range of clinically useful molecular assays. These novel applications could enable a true minimally invasive and broadly applicable “liquid biopsy” for cancer cells in the blood. Illustration by Nicole Wolf, MS, ©2025.

For more powerful but stringent single-cell approaches, CTC purification requires an additional 10^3^- to 10^4^-fold enrichment beyond most initial capture technologies (Figure 3). This additional purification typically involves labeling residual WBCs and CTCs using fluorescence-conjugated antibodies, optically identifying CTCs, and sorting them individually using one of a number of compatible cell sorting technologies; these include, but are not limited to, CMOS-driven Dielectrophoresis (DEPArray),^135^ droplet encapsulation and electrostatic sorting using microfluidic fluorescence-activated cell sorting (FACS),^15^ and image-guided automated cell picking (CellCelector).^73^ An alternative to single-CTC purification is the use of droplet-based single-cell RNA sequencing (scRNA-seq) technologies, which can process up to 10,000 cells per channel.^14^ However, very low CTC purity following initial enrichment remains a barrier.^136^

Either batch processing or microfluidic (in-line) technologies can sort CTCs from blood cells (Figure 3). Batch purification can be performed in a vial using common lab equipment. While more accessible, the washing and centrifugation steps from even simple debulking can lead to loss of very rare CTCs, resulting in reduced yield and purity.^137^ On the other hand, microfluidics devices operate in a continuous flow, whereby each CTC or blood cell individually enters the active sorting area, resulting in exceptional yield and purity of captured cells.^113^ However, many microfluidics devices are typically restricted to handling smaller volumes of whole blood, with highly specialized channels to process even 10 mL, let alone highly concentrated apheresis products (see below). Although current sophisticated microfluidic devices require a high level of expertise, it is anticipated that they may become more accessible as the technology reaches maturity, paralleling other technologies such as FACS or DNA sequencing equipment (many of which rely on microfluidic cartridges). Looking to the future, we expect that increasing applications of high blood volume CTC enrichment will lead to optimization of single-cell purification technologies for RNA, DNA and protein analyses.

### High blood volume CTC enrichment

The ultimate challenge to CTC isolation is biological, rather than technical: namely, the very rare number of CTCs present in a standard blood tube. A number of approaches have been explored to overcome this issue by increasing the volume of interrogated blood. For instance, indwelling EpCAM antibody-coated CTC-trapping guidewires or indwelling venous cannulation catheters have been tested,^138-140^ although flow kinetics within large blood vessels limit contact time of the device with flowing blood, and placement of the indwelling device is invasive. CTC concentrations or yields may also be higher in the central venous system draining a tumor, but also require invasive procedures to sample.^77,141-144^ While cannulating such tumor draining vessels during surgical tumor resection is achievable, these procedures are not readily performed in the ambulatory setting.

**Figure 4.**
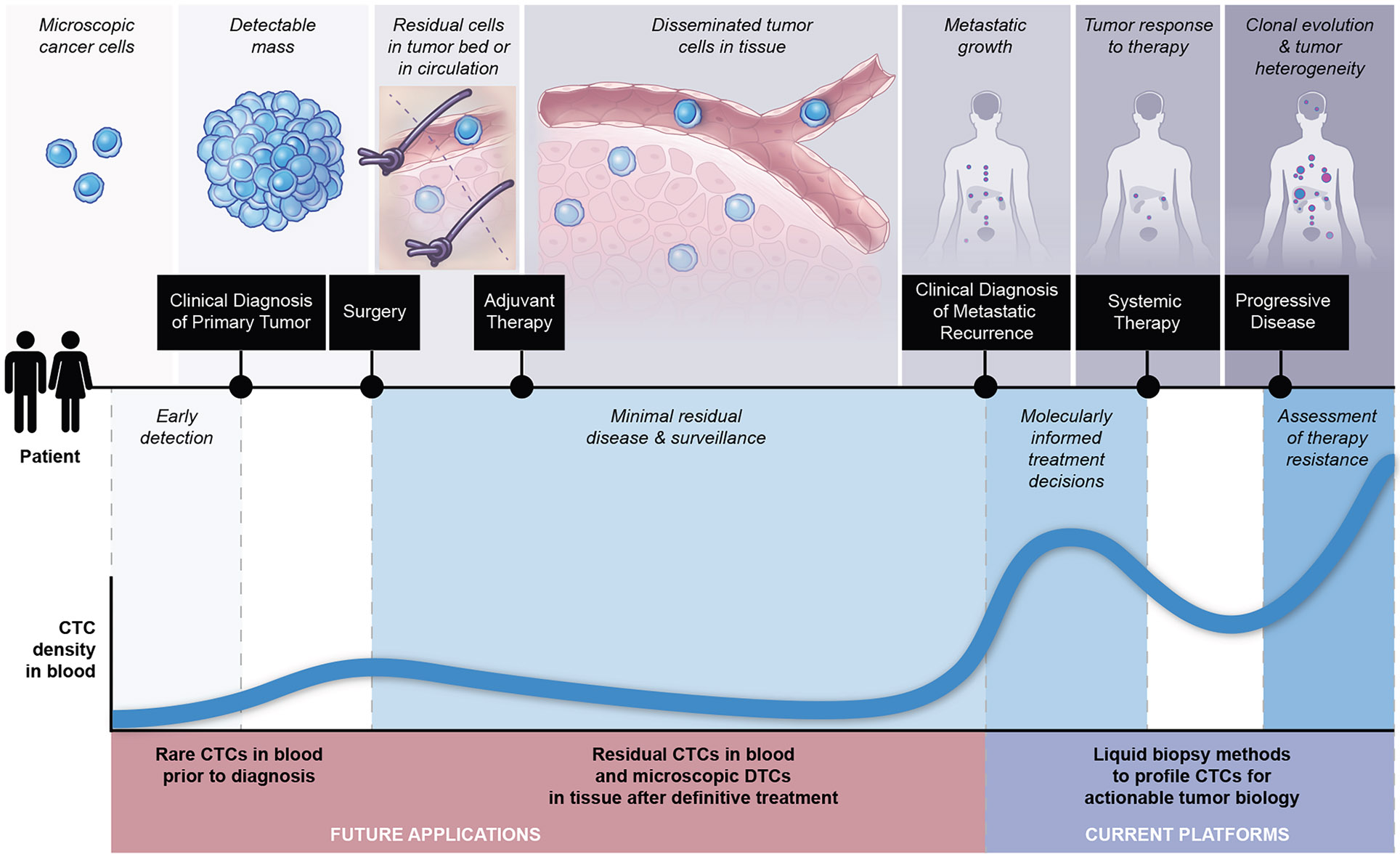
Potential applications of circulating tumor cells in the clinical management of cancer Advancements in CTC-based diagnostics may have important implications for both current and future applications of liquid biopsy-based clinical decision-making across the phases of cancer diagnosis and treatment. Illustration by Nicole Wolf, MS, ©2025.

Interrogating larger blood volumes has recently gained interest through the use of leukapheresis, a standard clinical procedure in which blood flows from one peripheral vein through an inline centrifuge that removes nucleated cells, returning plasma and RBCs through a contralateral vein.^15,19-22,138,145-147^ The entire blood volume can be processed over a few hours, with minimal loss of blood. While patients tolerate this procedure well if they have adequate peripheral venous access, the principal technical challenge has been subsequent analysis of massive numbers of highly concentrated nucleated blood cells. The CellSearch platform, even when used to maximal efficiency, can process only approximately 5% of a leukapheresis product.^14,15,147^ However, a recent WBC depletion microfluidic device, capitalizing on high-throughput flow rates and high-powered “magnetic lenses”, can sort through an entire leukapheresis product in one hour, with a capture efficiency of 85% and a final purity of ~0.1% (0.005–3.3%).^15,21^ Initial tests of leukapheresis products, derived from interrogation of 1–2 L of blood over 1 h and subjected to high-throughput microfluidic WBC depletion, have purified from 100–58,000 CTCs from individual patients with different types of metastatic cancer.^15,21^ This extraordinary yield thus makes a true “cell-based liquid biopsy” possible, enabling detailed CTC characterization and clinically relevant insights from a single patient (Figure 4). Leukapheresis is not routinely conducted in patients with solid tumors, and the requirements for good venous access, stable cardiovascular status, and access to a dedicated transfusion center, are logistically more challenging than a standard peripheral blood collection. However, it is possible that more accessible platforms that collect intermediate blood volumes will enable more routine clinical applications, and the information gained could even rival those of invasive tissue biopsies.

## MOLECULAR AND FUNCTIONAL BIOLOGY OF CTCs

### CTCs in metastasis

Metastasis comprises a complex series of stochastic events occurring in stepwise fashion in order for a tumor to establish a nascent metastatic lesion^148^ (Figure 1). This process begins with invasion and migration of tumor cells beyond the basement membrane, allowing for subsequent intravasation into blood vessels. Both cell intrinsic and extrinsic processes, as well as passive mechanical forces, can promote the shedding of tumor cells into circulation, giving rise to CTCs.^149^ These include multiple cell-autonomous factors, such as EMT, upregulation of TNF-α signaling or other pathways that disrupt endothelial junctions, or formation of invadopodia.^11,150-153^ Grouped migration of tethered epithelial cells, as opposed to single migratory mesenchymal cells, may also contribute to CTC clusters in the bloodstream.^72,154,155^ In mouse tumor models, transcriptional analyses in pancreatic cancer have suggested that many CTCs are derived from the tumor/stromal interface within the primary tumor,^156^ while studies of lineage-tagged cancer cells have pointed to hypoxic tumor regions as a predominant source of CTCs, with vascular endothelial growth factor (VEGF)-mediated changes contributing to their escape through endothelium.^157-159^ Tumor cell-extrinsic factors can also be instrumental in their intravasation, including interactions with perivascular macrophages.^160^ Once in the bloodstream, most CTCs travel as individual cells, but 1–30% may exist in multi-cellular clusters.^72,78,161^ Despite the burden of CTCs in the blood, only a minor population (by some estimates far less than 1%) develop into distant macrometastases.^162-165^ This reflects the fact that the blood circulation is highly unfavorable for CTC survival, given loss of matrix attachment (anoikis), high shear forces, high oxygen tension, and immune cell-mediated destruction during intravascular transit.^166-169^ Consistent with this, the average half-life of CTCs is short, on the order of seconds to minutes.^48-50,72,170^ For instance, in a mouse model where the vasculature of a tumor-bearing mouse was fused to that of a tumor-free littermate, CTCs appeared in the recipient mouse at intravasation rates anywhere between 60 and 107,000 CTCs per hour, where they persisted for only several minutes.^50^ In addition, a brief exposure to 1–2% of daily shed CTCs over a few hours was sufficient to generate macrometastases in the healthy recipient mouse, which were also capable of shedding their own CTCs from these metastases.^50^

The extravasation of CTCs from blood into distant tissues may be facilitated by their lodging and outgrowth from capillary beds.^77,171^ Leukocytes and platelet-derived interactions have also been implicated in facilitating extravasation into tissues.^172,173^ Upon exiting the circulation, these disseminated tumor cells (DTCs) require additional support from the stromal microenvironment to enable efficient growth into a metastatic colony.^25,174-177^ Such processes are thought to underlie the initial dormancy and delayed recurrence of metastatic cancer long after resection of a primary tumor, which for some tumors can even take place a decade or more after definitive surgery.^178-182^ While likely present in all tissues, DTCs have been best characterized in the bone marrow, due to accessibility for analysis.^183^ In fact, both CTCs in blood and DTCs in bone marrow have been reported in patients thought to have non-metastatic breast/prostate cancer, suggesting early microscopic dissemination.^67,178,182,184-187^ However, many patients with either CTCs or DTCs do not go on to develop overt metastatic disease, suggesting that additional factors are required to promote progression.^178,182^ Furthermore, DTCs are typically defined as cytokeratin-positive cells, but their genomic abnormalities, including the presence of CNV, have rarely been ascertained.^188,189^ Thus, the detection of either CTCs or DTCs in patients with localized cancer may identify risk, but do not by themselves indicate either the presence or the imminent emergence of metastatic disease.

Multiple signaling pathways between DTCs and local stromal cells have been described, including involvement of TGF-β ligands, NF-κB, thrombospondin-1, and interleukin signaling, all of which may dictate whether DTCs maintain a state of dormancy or initiate proliferation.^190-196^ In some cases, resident cells may also recruit neutrophils or other circulating cells that can facilitate tumor growth.^197^ On the other hand, immune surveillance has been implicated in maintaining DTC dormancy and suppressing initiation of metastatic proliferation.^198^ Taken together, these data suggest that metastasis is overall an inefficient process.^48,199-201^ This raises the possibility that identifying these early pioneering cells, at a time of very low micrometastatic burden, could identify patients who would benefit from escalated and potentially curative medical interventions.^181-183,202,203^ Our improved understanding of CTCs and their inherent biological properties in this phase of disease could thus have profound impacts on multiple steps of the metastatic cascade.

### Transcriptional profiling in CTCs

Recent molecular advances have revealed remarkable heterogeneity of CTCs isolated from the blood of patients with different types of cancer. New insights have emerged from antibody staining,^8,204-207^ *in-situ* RNA hybridization,^11,208^ bulk or single-cell DNA and RNA sequencing,^10,156,205,209-213^ and mass spectrometry/cytometry^18,214-217^ (Figure 3). These methods have since been applied to freshly isolated CTCs, as well as to *ex vivo* CTC cultures,^205,218-223^ enabling functional CRISPR screens and models of CTC-derived tumorigenesis and metastasis.^72,220,222,224,225^

The confirmation that cytokeratin-positive, CD45-negative blood cells are indeed tumor-derived was initially ascertained using antibody staining for lineage-specific markers—such as AR, PSA, and PSMA in prostate cancer^226,227^ or neural crest proteins in melanoma^90,130^—as well as using qPCR/ddPCR for panels of lineage-specific transcripts.^226,228,229^ Early studies using single-molecule RNA sequencing (smRNA-seq) of mixed cell populations identified CTC-specific transcripts, including non-canonical Wnt signaling outputs in pancreatic CTC-enriched cells.^10^ Further advances in CTC enrichment technologies made it possible to pick individual CTCs that were unfixed and identified using viable cell staining for surface marker expression,^63^ enabling high resolution scRNA-seq.^72,156,210^ Such studies have identified Wnt activation in castration-resistant prostate cancer (CRPC) and the presence of multiple distinct AR variants within single CTCs from individual patients.^88,210,226^ These methods have also revealed remarkable cross-sectional and spatiotemporal heterogeneity across CTCs; for instance, in hepatocellular carcinoma, scRNA-seq has revealed differences in transcriptional programs across different sampling sites, including processes that mediate EMT and immune invasion^230,231^; in prostate cancer, therapeutic inhibition of AR signaling leads to subpopulations of CTCs that upregulate non-canonical Wnt signaling to counteract AR blockade^210^; and in breast cancer, these approaches have uncovered different patterns of ER expression, divergent expression of Notch or HER2 as markers of chemoresistance versus cell proliferation, and expression of novel upregulated genes not previously associated with classical EpCAM+ CTCs.^205,232^ These findings have increased our understanding of CTC biology and may have clinical implications, for instance with elevated expression of both epithelial proteins and ribosomal proteins within single CTCs as a predictor of shortened survival.^225^

One caveat is that, given the small number of CTCs obtained from any single blood tube, most studies reflect an aggregate drawn from multiple patients, thereby confounding intra-patient and inter-patient heterogeneity. The development of CTC analyses from larger blood volumes can now overcome this challenge.^15^ With thousands of CTCs recovered from individual patients with metastatic cancer, scRNA-seq can now interrogate tumor heterogeneity within individual patients. As an example, in one patient with metastatic prostate cancer, scRNA-seq of 30 CTCs revealed two dominant subpopulations of cancer cells: half of CTCs had elevated expression of fibroblast growth factor receptor (FGFR) signaling, while the other half had marked elevation of inflammatory cytokines. In a second patient, the majority of 74 CTCs expressed high levels of classical AR target genes, MYC, and oxidative phosphorylation readouts, whereas 20% of CTCs lacked AR dependent genes but had high expression of neuroendocrine markers, indicating a clinically unsuspected lineage transition.^15^ Moreover, combined DNA/RNA sequencing applied to the same single cells identified small “double-negative” CTCs—staining for neither epithelial nor hematopoietic markers—as neuroendocrine-like cells, sharing identical clonal CNV markers with accompanying AR-driven CTCs, but expressing neural crest transcripts.^15^ Dual studies of DNA/RNA from single CTCs also provide definitive evidence that the great majority of circulating non-hematopoietic cells in the blood of metastatic cancer patients harbor CNV, the *sine qua non* of malignancy.^15^

### Mutational analysis and copy number variation

Mutational analysis of CTC-enriched populations was first used to document the emergence of the T790M mutation in the epidermal growth factor receptor (EGFR), which confers drug resistance in patients with EGFR-mutant non-small cell lung cancer (NSCLC).^233^ Additional studies reported the acquisition of *ESR1* (encoding estrogen receptor) mutations in patients with heavily treated metastatic breast cancer,^218,234^ *ALK* mutations in NSCLC,^235^ and *AR* alterations in prostate cancer.^88,92,210,236^ Such genotyping studies are now more readily performed using ctDNA sequencing,^237^ which are concordant for typing of *ESR1* or EGFR mutations with CTC-derived analyses in 40–90% of cases.^238,239^ Additionally, genome sequencing has permitted spatiotemporal tracking of widespread genetic changes in CTCs, both between and across patients, including gains and losses in copy number.^22,240-243^ Recent efforts have sought to increase sensitivity through whole-genome amplification-free methods, which could capture clinically relevant genomic information not necessarily evident from ctDNA or tumor tissue.^18^ Additionally, in a recent study of high blood volume CTC isolation, pooling CNV-confirmed CTCs enabled whole-exome DNA sequencing, with vastly improved mutation detection, compared with ctDNA.^15^ For such approaches, initial testing of individual candidate CTCs for the presence of tumor-defining CNV, then allows for either single cell or high purity pooled analysis for RNA or whole-exome sequencing analyses. High blood volume studies of CTCs are also unique in providing detailed clonal information about emerging subpopulations of cancer cells with distinct CNV or mutation profiles.^22^ Chromosome translocations with variable breakpoints may be more reliably detected using RNA sequencing from CTCs than from ctDNA,^244^ and FISH may demonstrate gene amplification/translocation.^65,245^ In addition, epigenome interrogation has revealed important features of cancer metastasis, including differential DNA methylation.^74^ This notably includes the ability to perform long-read DNA sequencing that is not possible with the short nucleosome-sized fragments obtained in ctDNA. These approaches have yielded novel categorical markers of cancer, such as the presence of Partially Methylated Domains (PMDs), which have important diagnostic and biological implications.^94^

### CTC heterogeneity and metastatic propensity

Single-cell genome sequencing applied to CTCs now makes it possible to more completely track spatiotemporal genetic changes, both within and across patients. As well-demonstrated by comparing matched primary versus metastatic lesions, the acquisition of metastasis-specific driver mutations does not routinely accompany the blood-borne dissemination of cancer.^119,240,246,247^ However, while CTCs frequently share mutational profiles with their tumor of origin, they may also display new mutations and copy number changes emerging during progression or development of drug resistance.^144,211,212,218,248,249^ In advanced disease, phylogenetic mapping studies support the notion that CTCs can also traffic between metastatic sites, adding to genetic complexity.^250-252^

In the absence of mutational drivers of metastasis itself, epigenetic mechanisms leading to altered transcriptional/translational outputs and cell lineages have been strongly correlated with metastatic propensity.^10,11,74,225^ For example, EMT grants epithelial-derived cancer cells certain migratory properties typical of mesenchymal cells, a process that is critical for normal embryogenesis and commandeered in cancer.^253,254^ CTCs may exhibit various degrees of plasticity with respect to expressed markers of epithelial or mesenchymal cell types, can share features of both cell types, or may upregulate canonical EMT transcriptional regulators.^11,13,60,123,223,229^ EMT is also associated with therapy resistance and the expression of mesenchymal markers and their transcriptional regulators within CTCs may vary dynamically, as individual patients respond and then progress on various therapies.^11,13,229^ Some CTCs may also express classical stem cell-like markers, such as CD44, CD133, BMI1, and ALDH1, possibly enhancing interconversion between cell states and adaptation to changing cellular environments, while disproportionately contributing to metastatic initiation.^220,223,255-257^ Furthermore, these stem-like features or other cell states of CTCs may be dynamic or reversible as they progress through changing environments.^258,259^ Similarly, cultured breast CTCs display remarkable plasticity, including spontaneous transitions between HER2-associated proliferation and Notch1-associated quiescence.^205^ Nonetheless, while these characteristics are intriguing, current evidence does not allow the categorical division of metastasis-competent and incompetent CTCs. In fact, a very small fraction of CTCs isolated from patient blood samples are capable of producing *ex vivo* cell lines (estimated at <5%), with the majority of CTCs imaged in the blood appearing to be at various stages of anoikis or apoptosis.^218^ Interestingly, once expanded in culture, CTC lines are capable of producing orthotopic tumors in immunosuppressed mice at very low inoculum, consistent with stem-like properties.^205,218^

### CTC and DTC-stromal interactions

The metastatic potential of CTCs is strongly influenced by cell-extrinsic interactions with stromal and immune cells. In a breast cancer model, lung tissue-derived bone morphogenic protein (BMP) ligands function as potent anti-metastatic signals, whose reversal is sufficient to drive metastasis formation.^193^ Communication between tumor cells and surrounding stromal cells may also be bidirectional; in a melanoma model, tumor-derived exosomes educate bone marrow progenitor cells to promote metastatic growth.^260^ Similarly, in a prostate cancer model, prolactin secretion by tumor cells triggers COX-2 production by neighboring stromal cells that subsequently support DTC growth.^261^ CTCs also can exhibit tissue tropism, driven by distinct tumor transcriptional programs and mediated by hypoxia signaling or by upregulation of other key pathways to counteract anti-metastatic stromal signals.^219,262,263^ Interestingly, established tumor cells can secrete chemokines that serve as attractants for other CTCs, facilitating tumor self-seeding once a metastatic site is established.^250^ While the interaction between CTCs/DTCs and the immune system is complex (see below), multiple studies in mouse models have highlighted the role of NK cells in suppressing early metastasis, suggesting that immune escape may partially drive metastatic progression.^198,264-266^ Finally, a fascinating feature of CTC biology has recently emerged from timed blood draws, suggesting that circadian rhythms may enhance CTC shedding at nighttime, possibly resulting from glucocorticoid or other hormonal factors that show oscillatory patterns.^267-270^ Taken together, different signaling pathways involving tumor cells, stromal cells within local tissues, as well as circulating factors may contribute to the ability of CTCs to initiate metastasis.

### CTC clusters and immune interactions

While most CTCs travel as single cells in the bloodstream, multi-cellular clusters increase in prevalence in advanced cancer and are correlated with adverse survival. The identification of CTC clusters is dependent upon the technologies applied to their enrichment, including size-based filters and microfluidic channels that selectively enhance capture of multicellular clusters.^65,99,134,271^ In mouse reconstitution experiments, CTC clusters display 25– to 50-fold increased metastatic potential compared with single CTCs, a phenomenon potentially attributable to reduced anoikis and improved survival upon landing in distant tissues.^72,154^ Mouse modeling using tagged cancer cells also demonstrates that CTC clusters are jettisoned from a single tumor deposit, rather than resulting from aggregation within the bloodstream or in CTC collection devices.^72^ Intercellular cell-cell adhesion and extracellular matrix molecules, including members such as plakoglobin, ICAM-1, CD44, and E-cadherin, play major roles in tethering CTCs and mediating *trans*-endothelial migration, as well as resistance to anoikis.^72,154,259,272-274^

While the majority of CTC clusters in human blood specimens are restricted to tumor cells, a subset are heterotypic, containing additional cell types including platelets,^275,276^ neutrophils,^73^ macrophages,^99^ fibroblasts,^71^ or myeloid-derived suppressor cells^277,278^ (Figure 1). For instance, CTCs may associate with tumor-associated fibroblasts in circulation, derived, at least in part, from the primary tumor, and permit efficient colonization of secondary destination sites.^71^ Furthermore, interactions between CTCs and non-malignant blood cells appear to be complex, with both pro-tumorigenic and anti-tumorigenic interactions noted. For instance, neutrophils can communicate with CTCs via integrins to increase tethering onto vascular endothelium in the liver,^279,280^ and more recently, scRNA-seq of CTC-neutrophil clusters has identified paired upregulation of several cytokines and corresponding receptors that confer a proliferative advantage to CTCs.^73^

In addition to being primary components of CTC clusters, hematopoietic cells can directly and indirectly modulate CTC-mediated tumorigenesis. Hematopoietic bone marrow progenitors can give rise to myeloid and monocytic subsets that prime the lung interstitia and condition the metastatic niche for CTCs.^281-285^ Extracellular traps released by neutrophils (so-called NETosis) can also sequester CTCs to enhance metastatic formation.^286-289^ In contrast, NK cells can target CTCs in the bloodstream, although platelet coating may suppress NK cell function or conceal them from NK cell recognition.^290-292^ Platelet coating may also present MHC class I molecules to NK cells, suppressing their recognition of MHC-deficient tumor cells.^291^ Finally, there has also been much interest in PD-L1 expression in CTCs, which ostensibly permits evasion from immune cells.^293,294^ Further characterization of CTCs and their interactions with the immune system may identify novel mechanisms to suppress these metastatic precursors.

### CTC-derived cell lines

The ability to propagate CTCs *ex vivo* has substantially enhanced our understanding of CTC biology.^218,295,296^ Such models are particularly useful in that they constitute patient-derived cultures, recapitulating both the initiating mutations present in the primary tumor, as well as those that are acquired during the course of therapy, as tumors initially respond and ultimately acquire drug resistance (Figure 4). *Ex vivo* models from CTCs have now been established across a variety of cancer types, including but not limited to breast,^218-220^ prostate,^221,297,298^ colon,^223,256^ gastroesophageal,^299^ lung,^222,300,301^ melanoma,^302^ head and neck,^303^ and pancreas cancer.^304^ While CTC cultures are infrequently established from standard blood specimens (approximately 5% success rate), high blood volume platforms enriching much larger number of CTCs may greatly improve on CTC culture success rates^23^ (Figure 3). Most CTC culture conditions include anchorage independent/low attachment culture, either in solution or within 3D scaffold-based systems, often under hypoxic culture conditions and using stem cell-like synthetic media, producing cell lines that persist indefinitely.^295,296^ Once established, CTC cultures are tumorigenesis-competent as xenografts in immunocompromised mice at low cell inoculum, although the time to tumor formation can be highly variable. Alternatively, direct inoculation of freshly isolated CTCs as xenografts without intermediate *in-vitro* expansion has been successful in some tumor types with abundant CTCs, such as small-cell lung cancer (SCLC).^305^ Interestingly, for many cultured breast cancer CTCs, intracardiac inoculation readily achieves distant metastases, whereas tail vein injection fails to generate proliferative lesions in the lung.^225^ This inefficiency has permitted experimental lentiviral CRISPR-mediated screens to identify modifiers of CTC-mediated metastasis, identifying ribosomal proteins and translational regulators as enhancers of metastasis, and a COX-2 degrader as a suppressor of metastasis.^225,306^

Cultured CTCs also provide unique opportunities for drug susceptibility testing within the genetic context of acquired mutations. For instance, breast CTCs harboring acquired mutations in PIK3CA and FGFR were shown to harbor synergistic drug sensitivity to inhibitors of both pathways in *in vitro* culture and mouse xenografts.^218^ Similarly, HER2 missense mutations, acquired by hormone-receptor positive breast cancers as they develop resistance to endocrine therapies, confer dramatic sensitivity to selective HER2 inhibitors.^307^ In contrast, only a small fraction of breast cancers with acquired mutations in BRCA1 or BRCA2 display sensitivity to PARP inhibitors.^308^ Functional studies of cultured CTCs thus enable susceptibility testing for new mutations acquired during the course of treatment and progression, including for those mutations detected by ctDNA whose functional significance in an individual patient may be uncertain.

## CTCs IN CLINICAL PRACTICE

For all successful diagnostic tests, clinical deployment is tightly linked to their impact on therapeutic interventions (Figure 4). As such, the co-development of CTC-based analytics and novel cancer therapies dictates the utility of CTCs in clinical practice. While ctDNA-based sequencing now plays a critical role in mutation-based targeted therapies, we envision a similar role for CTC analyses in the rapidly evolving world of antibody and T cell-based immunological therapies.^207,227^ Furthermore, interrogation of changes in gene expression via RNA and protein, chromatin alterations, or functional tumor cell properties may play increasing roles in cancer diagnostics, and the use of CTCs could even one day circumvent the need for invasive tumor biopsies.

### CTC monitoring in metastatic disease

High numbers of CTCs are generally correlated with adverse clinical outcomes, as demonstrated by early studies quantifying the presence of cytokeratin-positive cells in the blood of patients with metastatic cancer.^52,53^ In a landmark study in 2004, prospective enumeration of EpCAM+ CTCs using the CellSearch platform in patients with metastatic breast cancer treated with systemic therapy was shown to be independently prognostic for inferior progression-free survival (PFS) and overall survival (OS).^38^ This study used a validated cutoff of ≥5 CTCs per 7.5 mL of blood.^38,309^ These findings were later recapitulated across larger cohorts^310^ and in different cancer types, including colorectal cancer (CRC)^40^ and CRPC,^41^ leading to FDA clearance of the CellSearch platform for CTC enumeration as a prognostic marker in the clinical management of metastatic disease. The specific cutoffs associated with unfavorable prognosis notably vary across cancer types, from ≥3 CTCs/7.5 mL in CRC and renal cell carcinoma^40,47^ to ≥50 CTCs/7.5 mL in SCLC.^43^ These differences may reflect varying degrees of CTC shedding, inter-reader variability for image interpretation, as well as the variable efficacy of different therapeutic regimens.

Longitudinal monitoring of changes in CTC count appears to improve prognostication, with persistently elevated or rising CTC counts often heralding treatment resistance and declining counts signifying early treatment response^310,311^ (Figure 4). Importantly, CTC counts can independently stratify breast cancers into less aggressive versus more aggressive phenotypes, suggesting that CTC enumeration could provide even more granular staging and patient risk stratification than current clinicopathologic indices.^312^ CTC enumeration has also been studied as a surrogate biomarker to assess early treatment resistance warranting a switch in systemic therapy. However, early prospective studies have failed to show benefit based solely on CTC enumeration, without identifying specific vulnerabilities to guide subsequent-line therapies; for instance, neither the SWOG0500 nor the CirCe01 trials in metastatic breast cancer showed a survival benefit from non-directional switching to alternative cytotoxic chemotherapy regimens, prompted by persistently elevated CTC counts.^313,314^ On the other hand, the STIC CTC trial showed that an elevated CTC count may inform the choice of chemotherapy over endocrine therapies for metastatic ER+/HER2-breast cancer, although these findings are now less relevant with the advent of combined endocrine and CDK4/6 inhibitor therapy.^315,316^ All together, these studies, based on the prognostic power of CTC enumeration but without molecularly-informed predictions to guide alternative therapies, have limited the adoption of CellSearch in routine clinical practice.

Beyond enumeration, CTC analyses have also been applied in clinical trials using targeted molecular readouts. In breast cancer, the prospective COMETI trial examined whether a combined molecular index (CTC-ETI), consisting of CTC enumeration with expression testing for ER, BCL2, HER2, and Ki67, could predict responses to later-line endocrine therapy. However, CTC-ETI was not superior to CTC enumeration alone in identifying potentially endocrine-resistant tumors.^317^ Additional studies in breast cancer, including the DETECT III trial, have shown that in tumors without HER2 amplification, wild-type HER2 expression in CTCs does not predict an obvious benefit for the addition of the HER2 inhibitor lapatinib to standard treatment.^318,319^ In advanced prostate cancer, CTCs have been employed for detection of AR-V7, a variant of AR that maintains ligand-independent receptor signaling, despite AR pathway inhibitor (ARPI) therapy.^320^ Expression of AR-V7 in CTCs is strongly associated with resistance to ARPIs, but alternative therapies to which AR-V7+ prostate cancers exhibit increased sensitivity remain to be identified.^91-93^ Finally, in NSCLC, CTCs can capture the emergence of resistance mutations to tyrosine kinase inhibitors against EGFR^233,238^ and single-cell analysis have identified compound ALK-resistance mutations in patients with ALK-rearranged NSCLC.^235^ These studies point to the importance of linking specific molecular vulnerabilities identified in CTCs to actionable therapeutic interventions.

In comparison to other liquid biopsies, CTCs are most clearly suited for immunological therapies targeting proteins that are detectable on their cell surface. Studies of immune checkpoint inhibitors (ICIs) have tested the predictive value of PD-L1 expression on CTCs. However, the predictive value of PD-L1 expression in CTCs across different tumor types has to-date been inconsistent, as it has been for tumor biopsies themselves, given the multiple factors that contribute to general immune cell activation against tumors.^6-9^ More promising is the direct targeting of tumor epitopes using antibody-drug conjugates (ADCs), bispecific antibodies (BiTEs), targeted radioligands, or CAR-T cell therapies.^5,207^ In such cases, specific targeting requires “real-time” knowledge of targeted protein expression on tumor cells. While sampling a metastatic lesion is often considered to ensure that the targeted epitope is present on cancer cells, such invasive biopsies typically select a single relatively accessible metastasis, that may not be representative of the total tumor burden. In this context, CTCs may not only present a non-invasive alternative sampling approach, but as a summative representation of all tumor deposits, they may more clearly assess heterogeneity for the target of interest. Ongoing studies are aimed at refining and validating the predictive value of quantitative epitope expression.

### CTC detection in localized cancers

While rare, CTCs have potential to offer important insights into the early development of metastases, given current limits of detection for small metastatic lesions by modern radiographic modalities.^321^ This may be particularly relevant to high-risk early-stage disease or in the assessment of residual disease following curative intent surgery (Figure 4). For instance, CTCs have been detected in the blood of patients with early-stage localized cancers, including pre-operatively and following surgical removal of the primary tumor.^187,322-327^ Among patients with stage I-III breast cancer, approximately 20% may have detectable CTC events based on CellSearch criteria,^328^ and their presence is associated with an increased risk of recurrent disease and inferior overall survival.^87,181,182,328^ These observations thus raise two important questions: first, do these CTC events represent viable tumor cells with the capacity to develop into metastases; and second, can interventions at this stage of “minimal residual disease” (MRD) —where patients may have detectable CTCs, but no radiographic evidence of disease— alter the natural history to prevent recurrence and onset of progressive metastases that preclude cure? These questions have become increasingly relevant, as more systemic therapies shift toward peri-operative management of early-stage disease, presenting an opportunity for curative interventions that require risk-adapted approaches to balance benefit versus toxicity. For instance, in the phase III SUCCESS trial, the presence of CTCs prior to initiation and following adjuvant chemotherapy was associated with significantly worse disease-free and overall survival; approximately 18% of patients had persistent CTCs after two years of therapy, raising questions regarding the timing of more intensive surveillance or additional therapy prior to the detection of overt metastatic disease.^182^ Of note, an increasing number of clinical trials deploy ctDNA measurements of MRD after surgical resection of a primary tumor, with the goal of instituting therapy early, when the tumor burden is relatively low and curative therapy may still be possible. However, MRD measurements alone do not currently inform the selection of appropriate therapeutic choice. Concomitant CTC genetic analyses by ddPCR or next-generation sequencing could improve sensitivity of detection or identify actionable targets, an important area of future investigation.^329^ Along these lines, the German SURVIVE study will be the first large randomized trial to evaluate the survival benefit of liquid biopsy, including CTCs, for surveillance in early breast cancer as a trigger for restaging evaluations.^330^ This study will hopefully provide more definitive data on whether early interventions on MRD or oligometastatic cancer can improve long-term survival outcomes.

### Presymptomatic early cancer detection and screening

Observational studies have suggested that the presence of circulating tumor cells or non-viable cancer components are associated with elevated risk of a future cancer diagnosis^331-335^ (Figure 4). Current paradigms for blood-based screening of asymptomatic individuals for the presence of undetected cancer have focused on a number of ctDNA based assays, including detection of altered CpG methylation within short DNA fragments in the blood^336^ and differences in the sizes of DNA fragments derived from tumor cells, compared with normal blood cells (“fragmentomics” assays).^337^ These approaches have the advantage of high-throughput and low cost, but they suffer from a high rate of false-positive results when applied to a general population, where the true risk of cancer is low.^336,338^ Moreover, the tissue of origin for a suspected cancer identified by ctDNA is indirectly inferred from altered epigenetic patterns, whereas CTCs could provide direct transcriptional information about cell lineage. The complexity of CTC isolation and genetic analysis, however, currently precludes their use as a primary screening tool for cancer in a general population. Early attempts have included screening for CTCs in patients at high risk for lung cancer,^331^ as well as breast/prostate cancer.^333,334^ Future work is required to better define the role of CTCs in early detection, either as confirmatory testing following other screening methods or for application in particularly high-risk individuals.

### Therapeutically targeting CTCs

While much work has focused on the diagnostic utility of CTCs, there is also rationale for targeting these cells to suppress blood-borne metastasis. To date, the most compelling, albeit controversial, approach is through targeting platelets and suppressing COX-2 activity. COX-2 inhibitors and non-steroidal anti-inflammatory drugs (NSAIDS) have pronounced effects in suppressing primary tumorigenesis in colon and other types of cancers, but the associated thrombotic and cardiac risk have limited the enthusiasm for their use in cancer chemoprevention.^339^ Similarly, COX-2 inhibitors have pre-clinical and clinical efficacy in suppressing metastasis.^340-343^ Turning to more CTC-targeted approaches, a recent study tested the use of digoxin, which appears to suppress CTC-cluster formation and showed early promise in a small clinical trial.^74,344^ Of note, one direction that has failed due to associated clinical complications is the use of repeated apheresis to deplete CTCs from the circulation,^345^ a risky strategy that is inherently limited due to the short half-life of CTCs and their prompt regeneration from existing metastatic deposits. Finally, we note that antibody-driven therapies may be particularly effective in suppressing single CTCs in the bloodstream, which may have targetable epitopes, such as cadherin, that otherwise would be masked in organized tissues.^346^ While much remains to be done, such interventional studies hold the promise that such research on CTCs, in addition to its diagnostic utility, may ultimately enable suppression of metastatic disease.

## CLOSING REMARKS

CTCs underlie the lethal metastatic spread of cancer and have long been studied as a fascinating biological phenomenon, but challenges inherent in ultra-rare cell isolation from blood have so far precluded robust clinical applications. While technological innovations now introduce the possibility of novel diagnostic tools, these assays are simultaneously guided by emerging therapeutic needs. We envisage a time where isolation of sufficient numbers of CTCs from large blood volumes, using non-invasive automated technologies, will make it possible to routinely interrogate these cells for a wide array of clinically relevant analytes. In particular, the advent of highly effective cancer immunotherapy regimens —and their increasing diversity in targets— brings forth the potential need to identify the relevant cancer-associated proteins and real-time monitoring of targeted epitopes. These include a range of therapies, not limited to immune checkpoint blockade, bispecific antibodies, antibody-drug conjugates, antibody-radionuclides, and CAR-T and other cellular immunotherapies. Similarly, the emergence of resistant tumor subclones in patients may be identified by CTC analysis long before clinical relapse becomes evident, enabling earlier adjustments in therapy. This may also be relevant to patients with rising ctDNA levels following curative-intent surgery (MRD), in whom disease recurrence is expected, but optimal treatment choices may not be evident without comprehensive mutational sequencing of CTCs. Finally, we anticipate that pushing the envelope to identify *bona fide* CTCs in patients at high risk of cancer by virtue of ctDNA screening, suspicious radiographic lesions, or other genetic and environmental risk factors may contribute to the emerging tools for non-invasive cancer screening and early intervention.

Ultimately, understanding the intrinsic biological properties of CTCs may lead to effective anti-metastatic therapeutics, a field that has remained aspirational. The unique vulnerability of CTCs, including metabolic or survival pathways, as well as cell surface markers that are unique among cells within the bloodstream, may lead to highly specific therapeutic opportunities. At the same time, measuring CTC burden in the blood may identify individuals at risk for metastasis as well as quantify the response to therapeutic interventions to optimize potentially curative interventions. Taken all-together, the field of CTC analysis has brought together advanced bioengineering and microfluidics methods —focused on ultra-rare cell enrichment— with single-cell imaging, molecular diagnostics, and functional analyses. These innovative efforts aim to understand the process of cancer metastasis and employ the use of rare cancer cells in the blood to guide emerging clinical therapeutics. The convergence of these disciplines is now poised to shed light on a critical and potentially vulnerable node in the progression of cancer.
